# Multiplying the Stable Electrostatic Field of Electret Based on the Heterocharge‐Synergy and Superposition Effect

**DOI:** 10.1002/advs.202203150

**Published:** 2022-09-15

**Authors:** Shizhe Lin, Zisheng Xu, Shuting Wang, Jianglang Cao, Junwen Zhong, Guanglin Li, Peng Fang

**Affiliations:** ^1^ CAS Key Laboratory of Human‐Machine Intelligence‐Synergy Systems Shenzhen Institutes of Advanced Technology and Shenzhen Engineering Laboratory of Neural Rehabilitation Technology Shenzhen 518055 P. R. China; ^2^ Department of Electromechanical Engineering and Centre for Artificial Intelligence and Robotics University of Macau Macau SAR 999078 P. R. China; ^3^ Key Laboratory of Urban Rail Transit Intelligent Operation and Maintenance Technology and Equipment of Zhejiang Province College of Engineering Zhejiang Normal University Jinhua 321004 P. R. China

**Keywords:** electret, heterocharge‐synergy, non‐contact nanogenerator, stable electrostatic field, superposition effect

## Abstract

Owing to magic charge storage behavior, an electret can exhibit an external electrostatic field, which is widely used in numerous domains such as electronics, energy, healthcare, and environment. However, the theory of the charge storage mechanism still needs further development to enhance the performance and stability of the electret. Herein, a stable charge storage model known as the heterocharge‐synergy model (HSM) in electrets is proposed and verified, and the electrostatic field superposition effect of electrets is also proved. Based on the HSM and superposition effect, the stable electrostatic field intensity (average of ≈22.49 kV cm^−1^ and maximum of ≈29.58 kV cm^−1^, which is close to the minimum air breakdown field intensity of ≈30 kV cm^−1^) of the composite electret film is multiplied by simple layer‐by‐layer stacking. Utilizing the multilayer composite electret films and designing a two‐sided electrostatic induction structure, a two‐sided bipolar single‐electrode non‐contact nanogenerator is constructed with transferred charge density up to ≈132.61 µC m^−2^, which is twice as large as that of the non‐contact nanogenerators with one‐sided electrostatic induction structure. Clearing and utilizing the charge behaviors of the electret can boost the performance and enhance the stability of electret‐based devices in various domains.

## Graphical abstract

The heterocharge‐synergy model in electret is proposed and verified, and the electrostatic field superposition effect is also proved. Based on these charge behaviors, the multilayer composite electret film is constructed with a stable electrostatic field intensity (average of ≈22.49 kV cm^−1^ and maximum of ≈29.58 kV cm^−1^), which is close to the minimum air breakdown field intensity (≈30 kV cm^−1^).

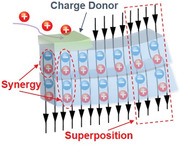

## 1 Introduction

An electret is a dielectric material that can quasi‐permanently store real charges (electrons or holes), which provide an external electrostatic field.^[^ ^1^ ^]^ Electrostatic fields can be used to construct numerous devices such as microphones,^[^ ^2^ ^]^ transistors,^[^ ^3^ ^]^ nanogenerators (NGs),^[^ ^4^ ^]^ wearable electronics,^[^ ^5^ ^]^ air filters,^[^ ^6^ ^]^ and drug delivery systems.^[^ ^7^ ^]^ Nevertheless, the electrostatic field of a normal electret is weak and easily affected by external temperature and humidity,^[^ ^8^ ^]^ resulting in poor working performance and stability of electret‐based devices. Thus, developing a theory of the charge storage mechanism and providing a universal and easy method for enhancing the performance and stability of electrets have great significance for its science and application.

**Figure 1:**
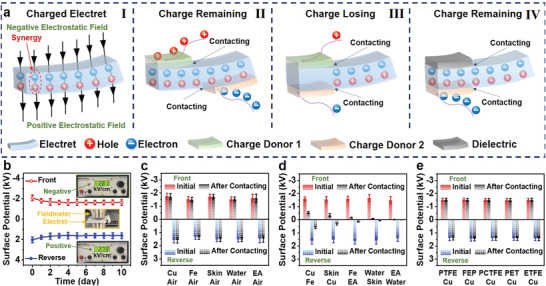
HSM of a single‐layer electret film. a) Schematic of the HSM. b) Surface potential characteristic of an SL‐PTFE over 10 days; and insets are fieldmeter. Initial and remaining surface potential of SL‐PTFE after contact with c) various charge donors on the front side, d) various charge donors on both sides simultaneously, and e) various dielectric films on the front side and Cu on the reverse side simultaneously. The thicknesses and size of all films (including SL‐PTFE and other dielectric films) are ≈30 µm and 4 × 4 cm^2^, respectively. 100 locations of a 3 × 3 cm^2^ sample were tested for obtaining the average surface potential. Error bars in all figures mean the standard deviation.

Currently, for an electret, it is generally believed that real charges are stored in the charge traps that exist on the molecular chains (particularly highly symmetric chains), between adjacent molecules, or at crystallite‐amorphous interfaces.^[^ ^9^ ^]^ The stability and quantity of charges are determined by the depth and number of the charge traps, respectively.^[^ ^1a^ ^]^ Moreover, during practical applications, electret materials inevitably come into contact with external objects (including solids, liquids, and gases), which causes physical phenomena, such as contact electrification^[^ ^10^ ^]^ and air breakdown,^[^ ^11^ ^]^ leading to the transfer or neutralization of charges. However, these theories cannot accurately explain the phenomenon of heterochargesynergy on different sides of the electret, as shown in **Figure** **1** in detail.

On the other hand, based on existing theories, numerous studies have been conducted to enhance the performance and stability of electrets. For instance, at the molecular chain level, the number of charge traps in an electret can be significantly increased by selecting an appropriate end group on the main chain molecules.^[^ ^12^ ^]^ At the crystallite‐amorphous interface level, charge traps can also be created by fabricating nanoscale interfaces between low‐crystalline and high‐crystalline regions.^[^ ^6a^ ^]^ Nevertheless, the processing methods of these studies must be adjusted according to the specific electret materials, thereby limiting their universal application. In addition, the electrostatic field intensities of these electrets are much smaller than the minimum breakdown field intensity of air (≈30 kV cm^−1^),^[^ ^13^ ^]^ see details in Discussion, Supporting Information.

Therefore, in this study, a stable charge storage model known as the heterocharge‐synergy model (HSM) for electrets, which can explain one‐sided contact‐stabilization phenomenon of the electret, was verified by testing the surface potential of the electret under various conditions and theoretically analyzing the charge behaviors of the electret. On the other hand, the electrostatic field superposition effect was proved and used to fabricate a multilayer electret film whose average and maximum electrostatic field intensity was risen to ≈22.49 and ≈29.58 kV cm^−1^, respectively. Based on the aforementioned charge behaviors, multilayer composite electret films (ML‐CEF) with high stability and electrostatic field intensity were constructed by avoiding two‐sided simultaneous contact of the electret and layer‐by‐layer stacking. Utilizing the inverse electrostatic field directions on different sides of the electret, a two‐sided bipolar single‐electrode non‐contact NG (TBSN‐NG) was designed using the ML‐CEFs, whose charge density is up to ≈132.61 µC m^−2^, 30.98 times that of the NG structured using a single‐layer composite electret film (SL‐CEF). Moreover, a two‐sided bipolar single‐electrode non‐contact rotatory disk NG (TBSN‐RDNG) was constructed by zooming in and refactoring the TBSN‐NG. Using the TBSN‐RDNG, a capacitor of 47 µF was charged to 50 V within 28.4 s. The facile and universal approach for enhancing the performance and stability of an electret by utilizing charge behaviors can be used for NGs and other electret‐based devices in various fields.

## 2 Results and Discussion

### 2.1 Heterocharge‐Synergy Model

It is well known that an effective charge injection method should be applied to allow the electret to obtain stable real charges before use.^[^ ^1a^ ^]^ In this study, negative corona charging was adopted to charge a polytetrafluoroethylene (PTFE) film, which is a normal commercial electret film that can easily trap real charges. During the process of negative corona charging, negative ions as the primary carriers transferred their electrons into charge traps on the front side of the PTFE electret film. Simultaneously, holes coming from the electrode and gap breakdown were injected into the charge traps on the reverse side of the PTFE film under an electric field (Figure S1, Supporting Information). In particular, the PTFE film was not adopted any treatment process except corona charging.

After charge injection, the surface potential and electrostatic field strengths of the single‐layer PTFE electret film (SL‐PTFE) on both sides exhibited approximately equal absolute values, but opposite directions (Figure 1aI,b and Table S1, Supporting Information). Specifically, the average surface potential of the SL‐PTFE on the front and reverse sides with almost the same variation trend remained stable at ≈about −1.635 and 1.618 kV, respectively, after 10 days. The electrostatic field strengths of the SL‐PTFE on the front and reverse sides were about −2.81 and 2.79 kV cm^−1^, respectively, which were tested by a fieldmeter (insets of Figure 1b). The almost identical variation trend and absolute value may indicate the interdependency between electrons and holes trapped in an electret. Moreover, the mirror symmetry of the surface potential distributions further reveals that there must be a synergy of trapped heterocharges (electrons and holes) on different sides of the electret (Figure S2, Supporting Information).

A series of experiments for observing charge behaviors have also been conducted to prove the synergy of heterocharges. First, as shown in Figure 1aII,c and Figure S3, Supporting Information, after contact with various charge donors (including copper [Cu], iron [Fe], skin, water, and ethyl alcohol [EA], see details in Discussion, Supporting Information) on the front side or reverse side separately, the surface potential of the SL‐PTFE on both sides hardly changed, indicating that electrons or holes in the electret will remain stable when one of the two types of charge is strongly attracted (including electrostatic attraction and contact electrification) separately. Second, as shown in Figure 1aIII,d and Figure S4a–e and Table S2, Supporting Information, after contact with any two of the charge donors (including Cu, Fe, skin, water, and EA) on both sides simultaneously, the surface potentials of the SL‐PTFE on both sides reduced significantly and synchronously (see more details in Discussion, Supporting Information), demonstrating that electrons and holes trapped in different sides of the electret will be lost synchronously when they are strongly attracted simultaneously. Finally, as shown in Figure 1aIV,e, after contact with various dielectric films, including PTFE, fluorinated ethylene propylene (FEP), polychlorotrifluroethylene (PCTFE), polyethylene glycol terephthalate (PET), and ethylene‐tetra‐fluoro‐ethylene (ETFE), on the front side and Cu on the reverse side simultaneously, the surface potentials of the SL‐PTFE on both sides hardly changed, verifying that electrons and holes trapped in different sides of the electret will remain stable when one of the two types of charge is strongly attracted and the other is weakly attracted simultaneously. The synergy of heterocharges in an electret, causing a one‐sided contact‐stabilization phenomenon (Figure 1aII,IV), as a stable charge‐storage model can be referred to as HSM. To verify the universality of the HSM, the surface potential characteristics of the other three normal electret films (FEP, ETFE, and PCTFE) were tested. Figure S5, Supporting Information, shows the surface potential characteristics of the single‐layer FEP, single‐layer ETFE, and single‐layer PCTFE after contact with various charge donors, which have the same variation trend as the SL‐PTFE.

### 2.2 Physical Phenomena in a Single‐Layer Electret Film

**Figure 2:**
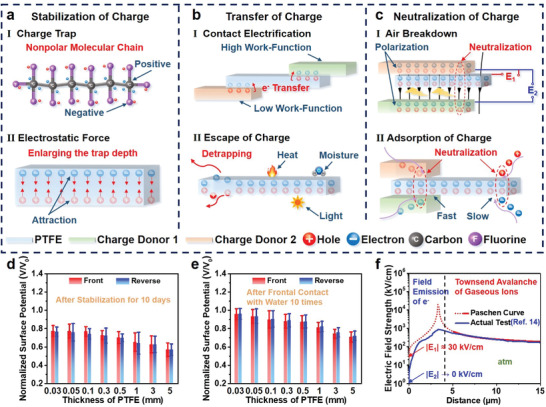
Physical phenomena in a single‐layer electret film. Schematic of the physical phenomena that cause the a) stabilization, b) transfer, and c) neutralization of charge, respectively. Surface potential characteristics of the SL‐PTFE with a series of thicknesses d) after stabilization for 10 days and e) after frontal contact with water ten times, respectively; *V* and *V*_0_ stand for test and initial value of surface potential, respectively. f) Characteristic curve of air breakdown. Actual test line (blue). Reproduced with permission.^[^ ^14^ ^]^ Copyright 1969, Springer Nature; atm stands for atmosphere. The size of each SL‐PTFE is 4 × 4 cm^2^. 100 locations of a 3 × 3 cm^2^ sample were tested for obtaining the average surface potential. Error bars in all figures mean the standard deviation.

To explain the HSM in detail, the physical phenomena that cause the stabilization, transfer, and neutralization of charges should be comprehensively analyzed. First, in the HSM, charge stabilization is due to the equal numbers of electrons and holes on different sides, which requires the electret material to have numerous positive and negative charge traps. For instance, the SL‐PTFE comprising non‐polar molecular chains can stably trap electrons and holes in different sides separately (**Figure** **2**aI and Figure S1 and S6a,b, Supporting Information). In contrast, a single‐layer polyvinylidene‐fluoride (PVDF) film comprising polar molecular chains hardly has real charge traps, which leads to a significant decrease of the surface potentials on both sides after negative corona charging for only 20 min (Figure S6a,b, Supporting Information). Moreover, the electrostatic force between the electrons and holes can enlarge the trap depth and make them more stable (Figure 2aII). However, the clear decrease in the surface potentials of the SL‐PTFE occurred as the thickness increased after stabilization for 10 days (Figure 2d and Figure S7, Supporting Information), because the intensity of electrostatic force is affected by the distance (thickness of the SL‐PTFE). In particular, the surface potentials of the front side are always a little higher than the reverse side, that is because the stability of electrons is higher than that of holes in the PTFE.

Second, as we know, the minimum energy required to transfer electrons from the interior of a material to the surface is the work‐function. In particular, electrons are usually transferred from a low work‐function material to a high work‐function material because of contact electrification during contact (Figure 2bI). Nevertheless, in the HSM, contact electrification is greatly suppressed and the transfer of charges hardly occurs in the SL‐PTFE when only one side is contacted (Figure 1aII), owing to the deep depth of the charge traps and the strong electrostatic force of heterocharges in different sides. Moreover, even though the electrostatic force is decreased as the thickness of the SL‐PTFE increased, the majority of the charges remain stable after repeated contact. Figure 2e and Figure S8, Supporting Information, demonstrate that the surface potential of the SL‐PTFE with a series of thicknesses (0.03 to 5 mm) became stable and mainly remained (front side: 95.8% to 71.0%; reverse side: 96.2% to 72.1%) after frontal with water ten times. A similar variation of the surface potentials of the SL‐PTFE with a series of thicknesses occurred after reversed contact with water ten times (Figure S9 and S10, Supporting Information). In particular, the surface potentials of the contact side are always a little lower than those of the non‐contact side after contact with water ten times, because the charges in the contact side are easier to escape than those on the non‐contact side. Additionally, some of the charges in shallower traps were slowly transferred into the air, leading to a decrease in the surface potential (Figure 2d), which is mainly caused by heat, moisture, and light in the surroundings (Figure 2bII).

Finally, air breakdown occurs, leading to the neutralization of charges during contact with charge donors which are easily polarized by the electrostatic field on the electret film and generate a new electric field (Figure 2cI). When only one side of the SL‐PTFE is close to a charge donor, there is an electric field (*E*_1_) between SL‐PTFE and the charge donor (Figure S11a,b, Supporting Information). The maximum of |*E*_1_| is ≈30 kV cm^−1^, which follows Paschen's law because only the Townsend avalanche of gaseous ions occurs between the SL‐PTFE and the charge donor (Figure 2f and Figure S12a–c, Supporting Information, see more details in the Discussion, Supporting Information).^[^ ^11^, ^14^ ^]^ When both sides of the SL‐PTFE were close to the charge donors, another electric field (*E*_2_) was generated between the two charge donors. The maximum of |*E*_2_| is ≈0 kV cm^−1^ because of the electrostatic attraction force from the charge donor (Figure 1d and Figure S4a–e, Supporting Information) and the field emission of electrons which occurs between two charge donors (Figure 2f and Figure S12b, Supporting Information).^[^ ^11^, ^14^ ^]^ When one side of the SL‐PTFE is close to the charge donor and the other side is close to the dielectric, the dielectric with a weak polarization intensity is polarized by the SL‐PTFE, generating a weak inverse electric field, and causes a weak shielding effect, which hardly affects the breakdown electric field intensity (Figure S11d, Supporting Information). Additionally, the surface potential characteristics of the SL‐PTFE covered with discharged PTFE with a series of thicknesses are shown in Figure S13b, Supporting Information, which can quantify the shielding effect of PTFE (Figure S13a, Supporting Information). When the electric field intensity of the SL‐PTFE is ≈1.635 kV cm^−1^ (Table S1, Supporting Information), air breakdown occurs and leads to charge neutralization only when both sides of the SL‐PTFE are close to the charge donors. Additionally, the electrostatic field of the SL‐PTFE, which causes the adsorption of charges coming from charge donors and surroundings, leads to the neutralization of electrons and holes simultaneously (Figure 2cII).

### 2.3 Superposition Effect of Electrostatic Field

Although the electrostatic field intensity of the SL‐PTFE (≈1.635 kV cm^−1^) is stable in the HSM, it is far from the maximum value of the breakdown electric field intensity (≈30 kV cm^−1^). Based on the weak shielding effect of PTFE film (Figure S13, Supporting Information), it can be inferred that constructing multilayer PTFE (ML‐PTFE) via simple layer‐by‐layer stacking can increase the electrostatic field of the electret to ≈30 kV cm^−1^. Considering air breakdown is likely to occur between the probe and ground metal plate of the electrostatic fieldmeter during testing, and the corresponding relationship between the superimposed surface potential and the electric field strength of the ML‐PTFE was linear (*R* ^2^ = 0.99995, Figure S14, Supporting Information), the surface potential was applied to evaluate the electrostatic field.

**Figure 3:**
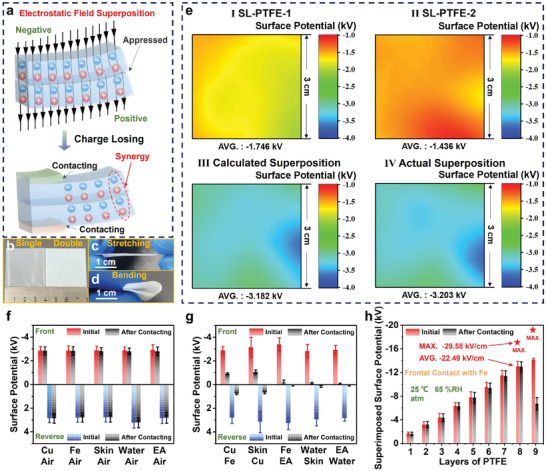
Superposition effect of the electrostatic field. a) Schematic of charge behaviors in the DL‐PTFE. b–d) digital images of the DL‐PTFE. e) Surface potential distribution images of I) the SL‐PTFE‐1, II) the SL‐PTFE‐2, III) calculated superposition of the SL‐PTFE‐1 and the SL‐PTFE‐2, and IV) actual superposition of the SL‐PTFE‐1 and the SL‐PTFE‐2. Initial and remaining surface potentials of the DL‐PTFE after contact with f) various charge donors on the front side and g) various charge donors on both sides simultaneously. h) Superimposed surface potentials of the ML‐PTFE with gradually increasing layers before and after frontal contact with Fe; MAX., AVG., and atm stand for maximum, average, and atmosphere, respectively. The size of each PTFE film is 4 × 4 cm^2^. 100 locations of a 3 × 3 cm^2^ sample were tested for obtaining the average surface potential. Error bars in all figures mean the standard deviation.

First, a double‐layer PTFE (DL‐PTFE) was constructed using two SL‐PTFEs (**Figure** **3**a,b). As shown in Figure 3e, the calculated and actual superposition surface potential distributions of the DL‐PTFE are almost identical, proving the idea of electrostatic field superposition. Moreover, the DL‐PTFE with excellent mechanical stability would not separate even after stretching and bending, owing to the strong electrostatic force between the two SL‐PTFEs (Figure 3c,d). In contrast, the DL‐PTFE, which can be simply regarded as a single unit, has similar charge behaviors as the SL‐PTFE. Specifically, the surface potentials of the DL‐PTFE on both sides had almost the same absolute value and variation trend over 10 days (Figure S15, Supporting Information). The surface potentials of the DL‐PTFE on both sides hardly changed after contact with various charge donors (including Cu, Fe, skin, water, and EA) on the front or reverse side (Figure 3f and Figure S16, Supporting Information). Furthermore, as shown in Figure 3g and Figure S17a–e, Supporting Information, the surface potentials of the DL‐PTFE on both sides decreased significantly and synchronously after contact with any two of the charge donors (including Cu, Fe, skin, water, and EA) on both sides simultaneously. Second, a triple‐layer PTFE (TL‐PTFE) film was also structured, where the electrostatic field superposition effect and charge behaviors in the HSM also existed (see details in Discussion and Figure S18–S21, Supporting Information). Finally, the superimposed surface potential characteristics of the ML‐PTFE with gradually increasing layers before and after frontal contact with Fe were tested (Figure 3h). The average and maximum absolute superimposed surface potentials of the ML‐PTFE with eight layers that can remain stable after frontal contact with Fe were about 13.067 and 17.187 kV, respectively. The average (∼22.49 kV cm^−1^) and maximum (∼29.58 kV cm^−1^) of electric field strength can also be calculated by a linear relationship (Figure S14, Supporting Information). Particularly, the superimposed surface potential of the ML‐PTFE with nine layers that decreased clearly after frontal contact with Fe must be caused by air breakdown, because its calculated maximum absolute value of electric field strength (∼32.58 kV cm^−1^) is greater than the minimum breakdown field intensity of air (∼30 kV cm^−1^). To verify the universality of the electrostatic field superposition effect, the superimposed surface potentials of the multilayer FEP, multilayer ETFE, and multilayer PCTFE with gradually increasing layers before and after frontal contact with Fe were tested, which have a similar variation trend to the ML‐PTFE (Figure S22, Supporting Information).

### 2.4 Structure and Charge Behaviors of the Composite Electret Film

**Figure 4:**
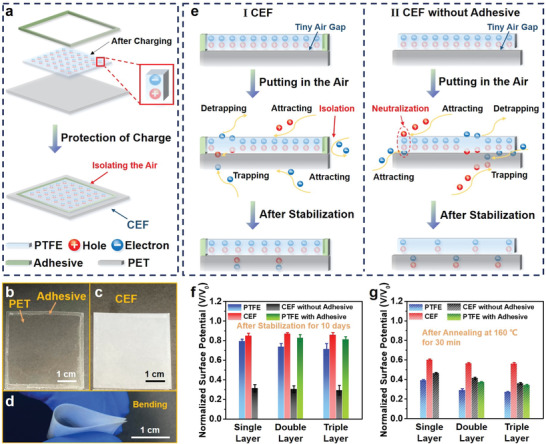
Structure and charge behaviors of composite electret film. a) Schematic of CEF. b–d) Digital images of CEF. e) Schematic of the physical phenomena in I) CEF and II) CEF without adhesive. The normalized surface potential of various electret films f) after stabilization for 10 days and g) after annealing at 160 °C for 30 min; *V* and *V*_0_ stand for test and initial value of surface potential, respectively. The size of each electret film is 4 × 4 cm^2^. 100 locations of a 3 × 3 cm^2^ sample were tested for obtaining the average surface potential.

Meanwhile, the stability of the electret can also be enhanced by utilizing the charge behaviors in the HSM. An SL‐CEF was easily constructed using an electret film (SL‐PTFE), a dielectric film (PET), and a certain amount of adhesive. The details of the fabrication process are presented in the Experimental Section. The SL‐CEF enhanced the charge stability by isolating the air and reverse side of the SL‐PTFE (**Figure** **4**a–c) and also exhibited excellent mechanical stability after bending (Figure 4d). In detail, for the SL‐PTFE without any contact, the transfer of charges is mainly caused by heat, moisture, and light in the surroundings (Figure 2bII), and the neutralization of charges is caused by the adsorption of electrons and holes coming from the surroundings in different sides simultaneously (Figure 2cII). The construction of the SL‐CEF can avoid the adsorption of electrons coming from the surroundings on the reverse side of the SL‐PTFE resulting in the enhancement of charge stability (Figure 4eI and Figure S23a, Supporting Information). In contrast, the construction of the SL‐CEF without adhesive cannot protect the charges and will accelerate the loss of charges because the PET will be polarized by the electrostatic field of the SL‐PTFE, trap electrons coming from the surroundings on the front side, and prompt the charge adsorption on the reverse side of the SL‐PTFE (Figure 4eII and Figure S24a, Supporting Information). As shown in Figure 4f,g and Table S3, Supporting Information, the ML‐CEFs (including SL‐CEF, double‐layer CEF, and triple‐layer CEF) exhibited higher stability than the corresponding ML‐PTFE, ML‐PTFE with adhesive, and ML‐CEF without adhesive both after stabilization for 10 days and after annealing at 160 °C for 30 min (see details in Discussion and Figures S23a–f and S24a,b, Supporting Information).

### 2.5 Structure, Working Principle, and Output of the Two‐Sided Bipolar Single‐Electrode Non‐Contact Nanogenerator

**Figure 5:**
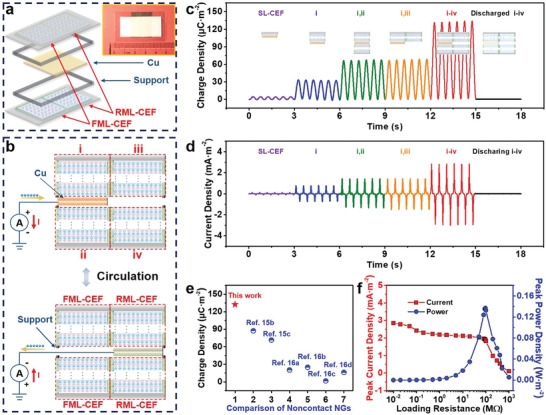
Structure, working principle, and output of the TBSN‐NG. a) Schematic of the TBSN‐NG, and the inset is a digital image. b) Working states of the TBSN‐NG during the periodic sliding cycles. Dynamic output c) charge and d) current curves of six types of NGs being constructed by different CEFs at a sliding speed of ≈8 cm s^−1^. The air gap between the slider and each stator determined by the support is ≈0.5 mm, and the effective area of the electrode is ≈4 cm^2^. e) Comparison of charge density between different non‐contact NGs.^[^ ^15^, ^16^ ^]^ Copyright 2 f) Matching impedance and output peak power of the TBSN‐NG at a sliding speed of ≈8 cm s^−1^.

In general, for a contact‐model NG, long‐time contact friction between different parts of the materials would cause interface heat loss and abrasion that seriously limits durability. In contrast, the non‐contact NG, mainly utilizing the electrostatic induction effect of materials, can avoid such drawbacks.^[^ ^15^ ^]^ However, the weak electrostatic field of existing materials seriously limits the output of a non‐contact NG. Herein, using front ML‐CEFs (FML‐CEFs) and reverse ML‐CEFs (RML‐CEFs) with the largest electrostatic field intensity (|*E*|  =  ≈22.49 kV cm^−1^), a TBSN‐NG was designed by utilizing different electrostatic field directions on the front and reverse sides of the electret in the HSM. The 3D structure diagram and the digital image of the TBSN‐NG are shown in **Figure** **5**a, which mainly comprise three parts: supports (PET), stators (FML‐CEFs and RML‐CEFs), and slider electrode (Cu). The air gap between the slider and each stator determined by the support was ≈ 0.5 mm, and the effective area of the electrode was ≈ 4 cm^2^. The details of the fabrication are presented in the Experimental Section and Figure S25a–e, Supporting Information. The working states of the TBSN‐NG during the periodic sliding cycles are shown in Figure 5b. In the process of moving the Cu from the space between two FML‐CEFs (i and ii) to the space between two RML‐CEFs (iii and iv), electrons were continuously injected into Cu resulting in the generation of a current that was directed from Cu to the ground. In contrast, when Cu moved from the space between iii and iv to the space between i and ii, electrons were continuously released from Cu resulting in the generation of a current that was directed from the ground to Cu. Considering the excellent structural design of the TBSN‐NG, increasing the layers of the CEF (i), constructing a two‐sided electrostatic induction structure (i, ii), and fabricating an alternating electrostatic field structure (i, iii) can enhance the output of the non‐contact NG. In particular, constructing a two‐sided electrostatic induction structure can significantly increase the theoretical transferred charge density of the non‐contact nanogenerator which is twice as large as that of constructing a one‐sided electrostatic induction structure (Figure S26, Supporting Information).

To ensure whether the practical performance of the TBSN‐NG is in line with expectations, the dynamic output charge density, current density, and open‐circuit voltage curves of the TBSN‐NG and the other five types of NGs were tested (Figure 5c,d and Figure S27, Supporting Information). The insets in Figure 5c show the specific constructions of various NGs. The transferred charge density, peak current density, and peak open‐circuit voltage of the TBSN‐NG (i–iv) are ≈132.61 µC m^−2^, ≈2.80 mA m^−2^, and ≈118.82 V at a sliding speed of ≈8 cm s^−1^, respectively, ≈30.98 times those of the NG constructed by an SL‐CEF. Additionally, the outputs of the NG (discharged i–iv) comprising discharged ML‐CEFs and Cu were tested. These facts prove that the high working performance of the TBSN‐NG is mainly owing to the strong electrostatic field of the ML‐CEFs. Moreover, the dynamic output charge curves of the TBSN‐NG in the contact mode (air gap close to 0) have been tested. As shown in Figure S28, Supporting Information, the maximum charge density of the TBSN‐NG in the contact mode is ≈156.29 µC m^−2^. Contact mode outputs higher transferred charges than the non‐contact model, that is because the air gap is smaller to induce a stronger electrostatic induction effect and most of the electrostatic charges remain during contact. A comparison of the charge density between the TBSN‐NG and various non‐contact NGs in recent studies is shown in Figure 5e and Table S4, Supporting Information,^[^ ^15^, ^16^ ^]^ indicating the excellent performance of the TBSN‐NG. The peak current and peak power densities of the TBSN‐NG at a sliding speed of ≈8 cm s^−1^ with various external load resistances are shown in Figure 5f, where a peak power density of 0.137 W m^−2^ was obtained at 95 MΩ.

### 2.6 Structure and Performance of the Two‐Sided Bipolar Single‐Electrode Non‐Contact Rotatory Disk Nanogenerator

**Figure 6:**
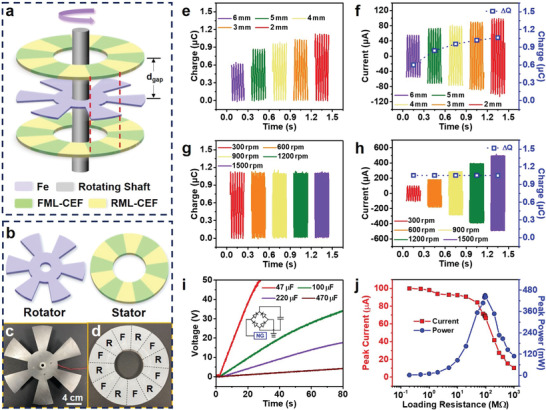
Structure and performance of the TBSN‐RDNG. Schematic of the a) TBSN‐RDNG and b) rotator, and stator. Digital images of c) rotator and d) stator; F and R stand for FML‐CEF and RML‐CEF, respectively. The effective area of each fan‐shaped ML‐CEFs is ≈20.94 cm^2^, and the distance between the rotator and stator (*d*_gap_) is adjustable. Dynamic output e) charge and f) current curves of the TBSN‐RDNG with a series of *d*_gap_ at a rotation speed of ≈300 rpm. Dynamic output g) charge and h) current curves of the TBSN‐RDNG with a *d*_gap_ of ≈2 mm for a series of rotation speeds. i) Voltage curves for charging capacitors with the TBSN‐RDNG at a rotation speed of ≈300 rpm, inset is a bridge rectifier circuit. j) Matching impedance and output peak power of the TBSN‐RDNG at a rotation speed of ≈300 rpm.

To further investigate the utility of the TBSN‐NG, a TBSN‐RDNG comprising a rotator (Fe, **Figure** **6**b,c) and two stators (FML‐CEFs and RML‐CEFs, Figure 6b,d) was constructed by zooming in and refactoring the TBSN‐NG (Figure 6a). The effective area of each fan‐shaped ML‐CEF was ≈20.94 cm^2^, and the distance between the rotator and stator (*d*_gap_) was adjustable. Due to the limitations of the manufacturing process, the minimum *d*_gap_ is ≈2 mm. The details of the fabrication process are presented in the Experimental Section. The working mechanism of the TBSN‐RDNG is similar to that of the TBSN‐NG.

Figure 6e,f shows the output charges, currents, and calculated transferred charges (Δ*Q*=∫*Idt*) of the TBSN‐RDNG with a series of *d*_gap_ at a rotation speed of ≈300 rpm. It can be seen from the results that the transferred charge (*Q*_max_), peak current (*I*_max_), and Δ*Q* increase (from ≈0.62 to ≈1.11 µC, from ≈56.0 to ≈101.3 µA, and from ≈0.60 to ≈1.06 µC, respectively) with a decrease in *d*_gap_ (from ≈6 to ≈2 mm), which is due to the enhancement of the electrostatic induction effect. In particular, the transferred charge density of the TBSN‐RDNG is ≈88.35 µC m^−2^, which is lower than that of the TBSN‐NG, because the *d*_gap_ is ≈2 mm. Moreover, the values of Δ*Q* are similar to those of *Q*_max_ for any *d*_gap_, verifying that the generation of current is mainly determined by the change in induced charges. In contrast, when the rotation speed increases from ≈300 to ≈1500 rpm with a *d*_gap_ of ≈2 mm, *I*_max_ increases from ≈101.3 to ≈585.2 µA (Figure 6h), but *Q*_max_ and Δ*Q* exhibit little change (Figure 6g,h), proving that the change in rotation speed only influences the time of charge transfer and has no effect on the number of transferred charges.

The TBSN‐RDNG was used to charge a series of capacitors to verify its utility. In detail, Figure 6i demonstrates that the capacitors of 47 and 470 µF can be charged to 50 V within 28.4 s and 4.46 V within 80 s, respectively, through a bridge rectifier circuit. In addition, the peak current and power of the TBSN‐RDNG at ≈a rotation speed of ∼300 rpm with various external load resistances are shown in Figure 6j, where a peak power of ≈0.451 W was achieved at 95 MΩ. Moreover, the TBSN‐RDNG also exhibited excellent durability, whose output *Q*_max_ was stable and remained at 97.3% even after ≈50 000 cycles (Figure S29, Supporting Information).

## 3 Conclusion

In conclusion, HSM was verified and used to explain the phenomenon of heterocharge synergy in electrets. The electrostatic field superposition effect of the layer‐by‐layer electret was also proved and utilized to structure an ML‐PTFE with a large average electrostatic field intensity of ≈22.49 kV cm^−1^, which is limited by the uniformity of the materials. Based on these charge behaviors, an ML‐CEF with high performance and stability was constructed by avoiding two‐sided simultaneous contact. A TBSN‐NG was fabricated using ML‐CEFs by constructing a two‐sided electrostatic induction structure and an alternating electrostatic field structure simultaneously. The charge density of the TBSN‐NG is up to ≈132.61 µC m^−2^, ≈30.98 times that of the NG structured by an SL‐CEF. In addition, a capacitor of 47 µF is charged to 50 V within 28.4 s using a TBSN‐RDNG that was constructed by zooming in and refactoring the TBSN‐NG. Moreover, clarifying charge behaviors, structuring the ML‐CEF, and modifying the processing technology to increase the uniformity of the electret materials can provide significant support for enhancing the performance and stability of the NGs and other electret‐based devices in various fields.

## 4 Experimental Section

### Fabrication of the ML‐PTFE

The SL‐PTFE (thickness of 30 µm) was purchased from Alibaba. The ML‐PTFE comprised corresponding numbers of SL‐PTFE by simple layer‐by‐layer stacking. Every SL‐PTFE was injected into charges by negative corona charging before stacking. The apparatus for corona charging comprised a high negative voltage power source (DW‐N503‐4ACDE), an array of corona needles, and a grounded metal plate. During negative corona charging, the SL‐PTFE was placed on a grounded metal plate 5 cm below the array of corona needles, and a voltage of −30 kV was applied between the array of corona needles and the grounded metal plate for 3 min.

### Fabrication of the ML‐CEF

The ML‐CEF was assembled using the corresponding ML‐PTFE, a PET dielectric film (thickness of 50 µm), and one of the adhesives (including epoxy, solid gum, and high‐temperature silicone sealant). The assembly process was divided into two steps. First, an ML‐PTFE and PET of the same size were stacked on top of each other. Second, the four sides of the stacked composite film were then sealed with a certain amount of adhesive.

### Fabrication of the TBSN‐NG

As shown in Figure S25a, Supporting Information, the components of the TBSN‐NG included four ML‐PTFE (eight layers, total thickness of ≈0.25 mm, and size of 2 × 2 cm^2^), two PET films (thickness of ≈0.5 mm and size of 3 × 5 cm^2^), two hollow rectangular PET films as supports (thickness of ≈0.75 mm, outer size of 3 × 5 cm^2^, and inner size of 2 × 4 cm^2^), a certain amount of adhesive (solid gum), and Cu foil as an electrode (thickness of ≈0.15 mm and size of 2 × 2.5 cm^2^). The Cu electrode was connected to a wire while it was working. The fabrication process is shown in Figure S25b–e, Supporting Information. After fabrication, the distance between the Cu electrode and each ML‐PTFE was ≈0.5 mm.

### Fabrication of the TBSN‐RDNG

The TBSN‐RDNG mainly comprised two identical stators and one rotator. The stator fabrication process was divided into two steps. First, an acrylic plate was cut into a disk as the base (thickness of ≈3 mm, inner diameter of ≈11 cm, and outer diameter of ≈21 cm). A circular hole in the middle was used to place the rotating shaft. Second, a set of complementary fan‐shaped‐array ML‐CEFs (radial angle of ≈30°, inner diameter of ≈11 cm, and outer diameter of ≈21 cm) was attached to the surface of the base using adhesive. Six FML‐CEFs and six RML‐CEFs were arranged alternately (Figure 6b,d). The effective area of each ML‐CEF was ≈20.94 cm^2^. A laser cutter was used to cut the rotator. The shape of the rotator (Fe, thickness of ≈0.5 mm) is shown in Figure 6b,c. The size of the six fan‐shaped parts of the rotator was the same as that of the fan‐shaped ML‐CEF.

### Characterization

The surface potential characteristics of the charged samples were detected using a non‐contact electrometer (Trek, model P0865) and a 2D slide platform (Pegasus Instrument Inc., model PG201W). The electrostatic field strength of the ML‐PTFE was tested using an electrostatic fieldmeter (Monroe Electronics, model 257D). PTFE films (a series of thicknesses) and various dielectric films (FEP, PCTFE, PET, ETFE, and PVDF; thicknesses of ≈30 µm) were purchased from Alibaba. The study protocol was thoroughly reviewed and approved by the ethical committee of the University of Macau (approval number BSERE21‐APP022‐FST). Informed signed consent for the volunteer tests was obtained from all volunteers prior to their participation in this study.

### Statistical Analysis

1) Normalized surface potentials (*V*/*V*_0_) in all figures were calculated by test surface potentials (*V*) and initial surface potentials (*V*_0_). 2) Error bars in all figures mean the standard deviation. 3) The size of all films (including electret and dielectric films) used for obtaining the surface potential was 4 × 4 cm^2^. 100 locations of a 3 × 3 cm^2^ sample were tested for obtaining the average surface potential. 4) When both testing the surface potentials of the front and reverse sides in all figures, the test locations were in two sides of the same area of the same films.

## Conflict of Interest

The authors declare no conflict of interest.

## Author Contributions

S.L., J.Z., G.L., and P.F. provided the design for the experiment and the implementation steps. S.L., S.W., and J.C. fabricated the device and measured the output performance. S.L. and Z. X. plotted and analyzed data. S.L., J.Z., G.L., and P.F. wrote the manuscript. Z.X. and S.W. provided some advice. All authors contributed to the manuscript.

## Supporting information

Supporting Information

## Data Availability

The data that support the findings of this study are available in the supplementary material of this article.
