# Supplementary material for: Multiplying the Stable Electrostatic Field of Electret Based on the Heterocharge‐Synergy and Superposition Effect
Source: Adv Sci (Weinh). 2022 Sep 15;9(32):2203150. doi: 10.1002/advs.202203150 (PMC9661856; doi:10.1002/advs.202203150)
Supplement: Supplementary file 1 — Supporting Information [file ADVS-9-2203150-s001.pdf]

ADVANCED SCIENCE
Open Access

Supporting Information

for *Adv. Sci.*, DOI 10.1002/advs.202203150

Multiplying the Stable Electrostatic Field of Electret Based on the Heterocharge-Synergy and Superposition Effect

Shizhe Lin, Zisheng Xu, Shuting Wang, Jianglang Cao, Junwen Zhong, Guanglin Li* and Peng Fang**

Supporting Information

Multiplying the Stable Electrostatic Field of Electret Based on the Heterocharge-Synergy and Superposition Effect

Shizhe Lin, Zisheng Xu, Shuting Wang, Jianglang Cao, Junwen Zhong, Guanglin Li,** and *Peng Fang**

S. Lin, S. Wang, J. Cao, G. Li, P. Fang
CAS Key Laboratory of Human-Machine Intelligence-Synergy Systems, Shenzhen Institutes of Advanced Technology & Shenzhen Engineering Laboratory of Neural Rehabilitation Technology, Shenzhen 518055, P. R. China.
E-mail: gl.li@siat.ac.cn, peng.fang@siat.ac.cn

J. Zhong
Department of Electromechanical Engineering and Centre for Artificial Intelligence and Robotics, University of Macau, Macau SAR, 999078, P. R. China.
E-mail: junwenzhong@um.edu.mo

Z. Xu
Key Laboratory of Urban Rail Transit Intelligent Operation and Maintenance Technology & Equipment of Zhejiang Province, College of Engineering, Zhejiang Normal University, Jinhua 321004, P. R. China.

Supplementary Discussion

Air Breakdown Field Strength

In a uniform electric field, the air breakdown electric field strength (E_{air}) can be calculated by empirical **Formula S1** and **Formula S2**.

$$U_b = 24.22\delta d + 6.08(\delta d)^{1/2} \text{ (kV)} \tag{S1}$$

$$E_{air} = U_b / d \tag{S2}$$

where, the air breakdown voltage is U_b, relative air density is δ, and gap distance is d.

The detailed derivation process can be seen in a book (J. M. Meek and J. D. Craggs, in *Electrical breakdown of gases*, Wiley, United States, **1978**). In contrast, in a non-uniform electric field using a sphere electrode, the minimum air breakdown field strength is also ~30 kV/cm (*J. Electrical Systems* **2012**, 8-2, 209-217).

Introduction of Charge Donors.

Charge donors are materials that can supply and transfer charges rapidly. Common charge donors include solid conductors (including metal and skin) and polar liquids (including water and EA). In particular, dielectrics hardly transfer charges in their natural state because of their high insulating properties, and non-polar liquids cannot supply charges for charge neutralization, as demonstrated in a previous study (Nano Energy, 2021,81:105618).

Synchronous Reduction of the Surface Potential of SL-PTFE on Two Sides.

As shown in **Figure 1a**-III, d, **S4a–e**, and **Table S2**, after contact with any two of the charge donors (including Cu, Fe, skin, water, and EA) on both sides simultaneously, the reduction values of the surface potential of the SL-PTFE on both sides were equal. The reduction value

must be determined by the size of the real contact area between the SL-PTFE and various charge donors during contact. A comparison of the real contact area between the five charge donors is shown in **Formula S3**, which must be influenced by the hardness and surface energy of the materials.

$$Cu \approx Fe < skin < water \approx EA \tag{S3}$$

For instance, as shown in **Figure 1d** and **Table S2**: (1) The front and reverse average surface potentials of the SL-PTFE remained at 34.8% and 36.4%, respectively, after contact with Cu on the front side and Fe on the reverse side simultaneously. (2) The front and reverse average surface potentials of the SL-PTFE remained at 21.8% and 21.9%, respectively, after contact with the skin on the front side and Cu on the reverse side simultaneously. (3) The front and reverse average surface potentials of the SL-PTFE remained at 10.3% and 9.6%, respectively, after contact with Fe on the front side and EA on the reverse side simultaneously. (4) The front and reverse average surface potentials of the SL-PTFE remained at 4.0% and 3.8%, respectively, after contact with water on the front side and skin on the reverse side simultaneously. (5) The front and reverse average surface potentials of the SL-PTFE remained at 0.5% and 0.4%, respectively, after contact with the EA on the front side and water on the reverse side simultaneously.

Paschen's Law.

Paschen's law was widely used to analyze air breakdown under a high electric field. Considering there is a high electrostatic field in the air gap between the PTFE electret film and charge donors, Paschen's law is well suitable for whether air breakdown occurs.

The critical breakdown voltage (V_b) can be described by a simple law (M.-A. Lieberman and A.-

J. Lichtenberg, in P*rinciples of Plasma Discharges and Materials Processing*, John Wiley & Sons, **2005**):

$$V_b = \frac{Bpd}{\ln(Apd) - \ln[\ln(1+\frac{1}{\gamma_{se}})]} \quad \text{(S4)}$$

and the critical breakdown electric field (E_b) can be described as:

$$E_b = \frac{Bp}{\ln(Apd) - \ln[\ln(1+\frac{1}{\gamma_{se}})]} \quad \text{(S5)}$$

where p is the gas pressure, d is the air gap, γ_{se} is the secondary electron emission coefficient, A is the saturation ionization in the air at a particular electric field pressure, and B is related to the excitation and ionization energies.

Charge Behaviors of TL-PTFE.

As shown in **Figure S18a–c** and **e**, the value of the actual superposition surface potential of the TL-PTFE was approximately equal to the sum of the values of the surface potential added by SL-PTFE-1, SL-PTFE-2, and SL-PTFE-3. The calculated and actual superposition surface potential distributions of the TL-PTFE are almost identical (**Figure S18d–e**), proving the ideal nature of electrostatic field superposition. In contrast, the TL-PTFE, which can be simply regarded as a single unit has similar charge behaviors to the SL-PTFE and the DL-PTFE. Specifically, the average surface potentials of the TL-PTFE on the front and reverse sides have almost the same absolute value and variation trend over 10 days (**Figure S19**). The surface potential of DL-PTFE on the two sides hardly changed after contact with various charge donors (including Cu, Fe, skin, water, and EA) on the front side or reverse side (**Figure S20a–b**). Furthermore, as shown in **Figure S17a–e**, after contact with any two of the charge donors (including Cu, Fe, skin, water, and EA) on the front and reverse sides simultaneously, the surface potentials of the DL-PTFE on different sides decreased significantly and synchronously.

Comparison of Charge Stability among Various Electret Films (Table S3).

(1) SL-PTFE, SL-CEF, and SL-CEF without adhesive

After stabilization for 10 days, the surface potentials of the SL-PTFE, SL-CEF, and SL-CEF without adhesive became stable and remained at 79.5%, 84.9%, and 31.4%, respectively (**Figure S23a**). In particular, the stability of the SL-CEF is better than that of the SL-PTFE because the PET in SL-CEF can avoid the adsorption of electrons coming from the surroundings on the reverse side of the SL-PTFE. In addition, the stability of the SL-CEF without adhesive became worse because the PET in the SL-CEF without adhesive was polarized by the electrostatic field of the SL-PTFE, trapped electrons coming from the surroundings in the front side, and prompted the charge adsorption of the SL-PTFE on the reverse side (**Figure 4e**-I and **Figure S24a**).

However, after annealing at 160 ^{o}C for 30 min, the surface potentials of the SL-PTFE, SL-CEF, and SL-CEF without adhesive became stable and remained at 38.9%, 60.0%, and 46.3%, respectively (**Figure S23d**). The stability of the SL-CEF still showed the best. Moreover, the stability of the SL-PTFE is worse than that of the SL-CEF without adhesive because the PET in the SL-CEF without adhesive also has some effect on isolating air, and the polarization level of the PET is low during the annealing process. Thus, the loss of charges in the SL-CEF without adhesive was mainly caused by the detrapping of charges during the annealing process (**Figure 4e**-II and **S24b**).

(2) DL-PTFE, DL-CEF, DL-CEF without adhesive, and DL-PTFE with adhesive

After stabilization for 10 days, the surface potentials of the DL-PTFE, DL-CEF, DL-CEF without adhesive, and DL-PTFE with adhesive became stable and remained at 73.9%, 86.9%, 43.5%, and 82.8%, respectively (**Figure S23b**). The quality comparison of the DL-PTFE,

DL-CEF, and DL-CEF without adhesive is the same as that of the corresponding single-layer electret films. Moreover, the stability of the DL-PTFE with adhesive is better than that of the DL-PTFE because the adhesive can isolate the air and decrease the loss of charges in the middle of the two SL-PTFE.

In contrast, after annealing at 160 ^{o}C for 30 min, the surface potentials of the DL-PTFE, DL-CEF, DL-CEF without adhesive, and DL-PTFE with adhesive became stable and remained at 29.0%, 56.4%, 41.3%, and 37.2%, respectively (**Figure S23e**). The quality comparison of the DL-PTFE, DL-CEF, and DL-CEF without adhesive is the same as that of the corresponding single-layer electret films. Moreover, the stability of the DL-PTFE with adhesive is worse than that of the DL-CEF without adhesive, indicating that the detrapping of charges is more significant than the neutralization of charges during the annealing process (**Figure 4e**-II and **S24b**).

(3) TL-PTFE, TL-CEF, and TL-CEF without adhesive, and TL-PTFE with adhesive

After stabilization for 10 days, the surface potentials of the TL-PTFE, TL-CEF, TL-CEF without adhesive, and TL-PTFE with adhesive became stable and remained at 71.1%, 85.9%, 29.2%, and 81.4%, respectively (**Figure S23c**).

In contrast, after annealing at 160 ^{o}C for 30 min, the surface potentials of the TL-PTFE, TL-CEF, TL-CEF without adhesive, and TL-PTFE with adhesive became stable and remained at 27.1%, 56.0%, 35.8%, and 34.2%, respectively (**Figure S23f**). Moreover, the quality comparison of the various triple-layer electret films was the same as that of the corresponding double-layer electret films.

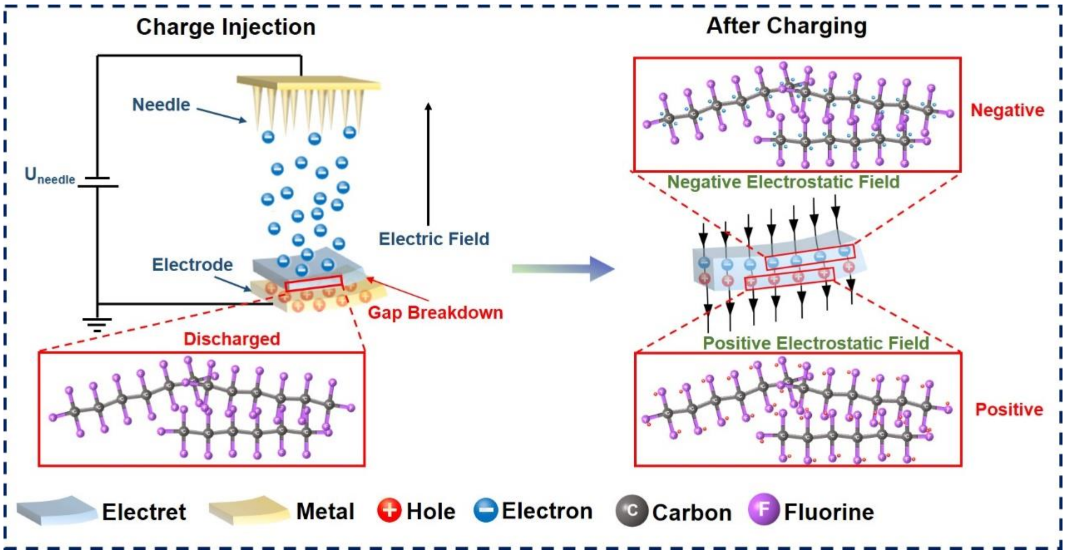

Figure S1. The schematic of the charge injection for an electret film.

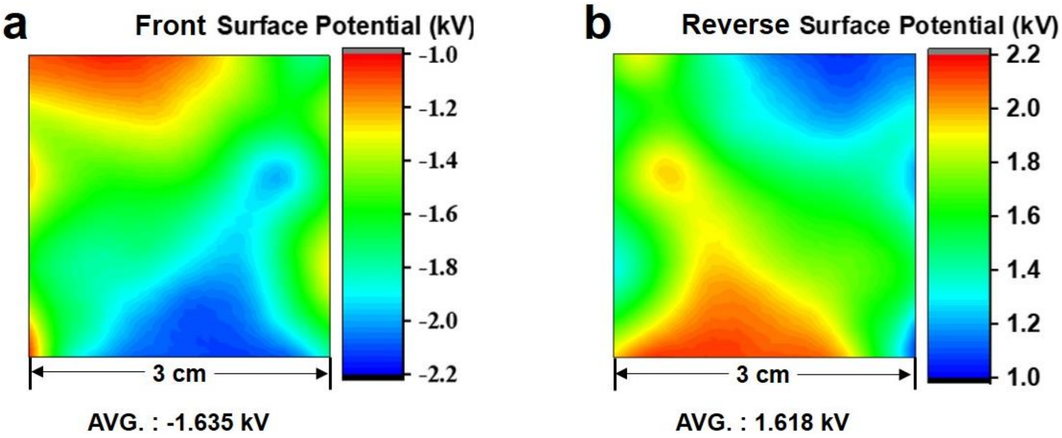

Figure S2. The surface potential distribution images of the SL-PTFE on a) the front side and b) the reverse side.

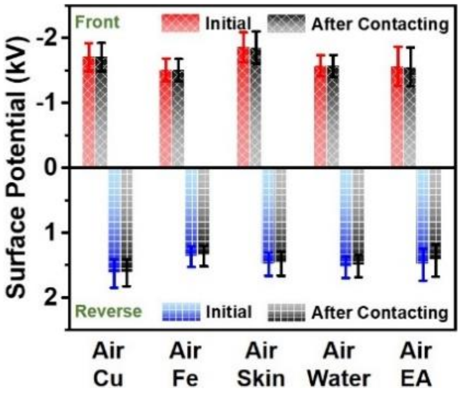

Figure S3. Initial and remaining surface potentials of the SL-PTFE after contact with various charge donors on the reverse side.

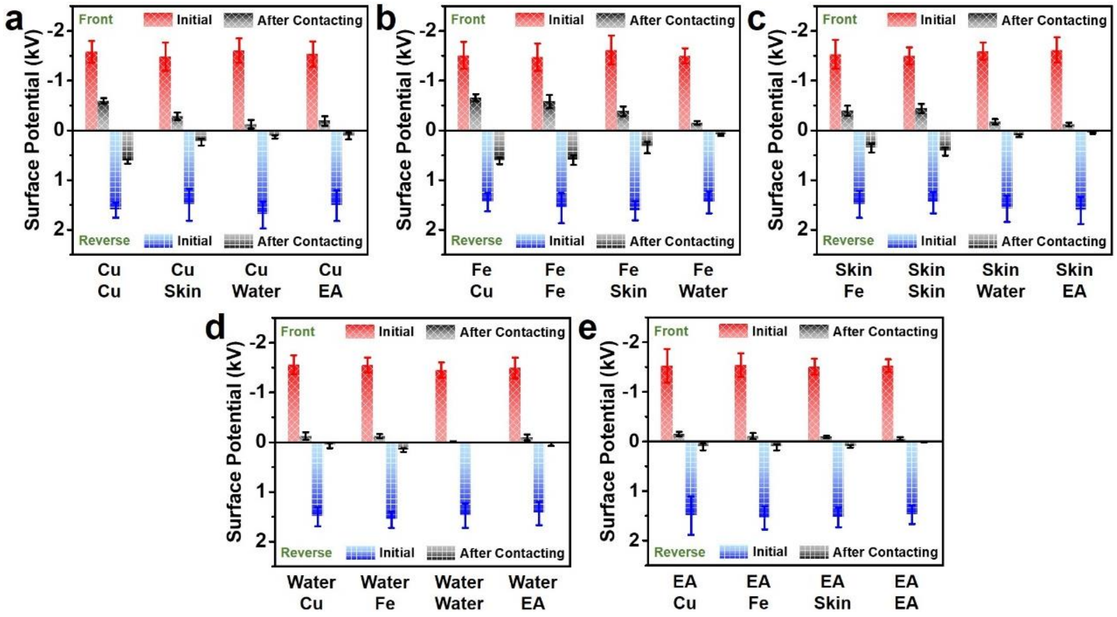

Figure S4. a-e) Initial and remaining surface potentials of the SL-PTFE after contact with various charge donors on the front side and reverse side simultaneously.

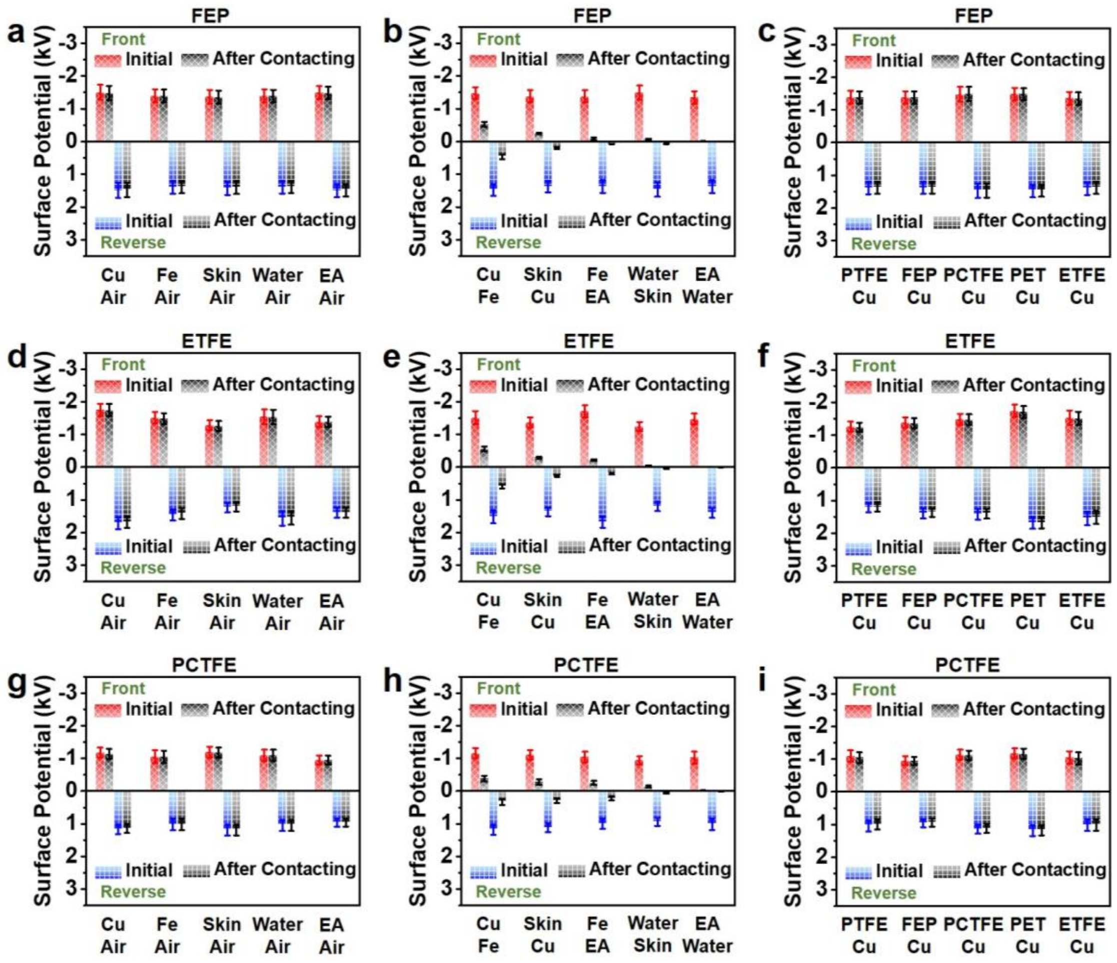

Figure S5. Initial surface potentials and remained surface potentials of the SL-FEP, SL-ETFE, and SL-PCTFE after contact with **a)**, **d)**, and **g)** various charge donors on the front side, **b)**, **e)**, and **h)** various charge donors on both sides simultaneously, and **c)**, **f)**, and **i)** various dielectric films on the front side and Cu on the reverse side simultaneously, respectively.

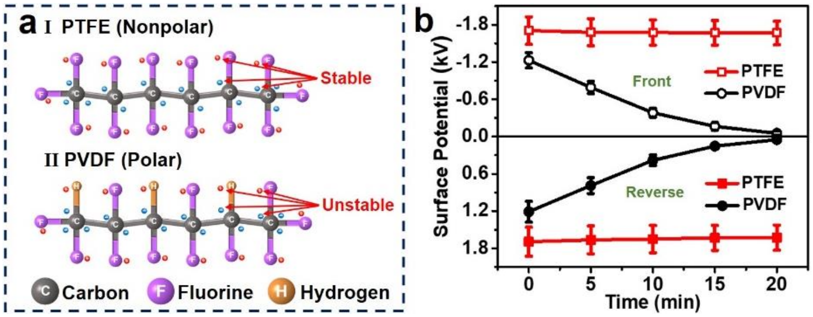

Figure S6. **a)** Schematic of the PTFE and PVDF. **b)** Surface potential characteristics of the SL-PTFE and SL-PVDF over 20 min.

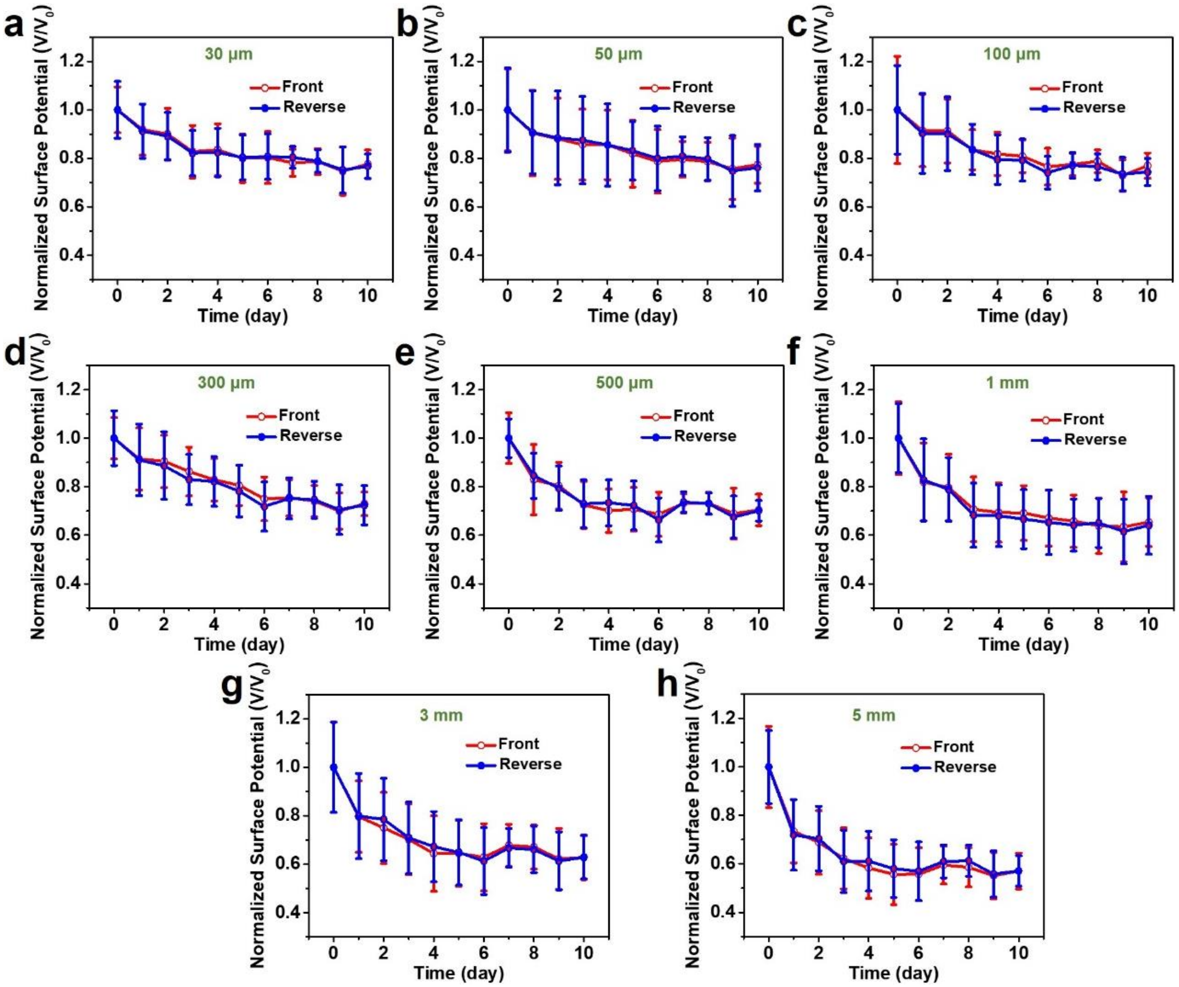

Figure S7. Surface potential characteristics of the SL-PTFE with a series of thicknesses over 10 days; V and V_0 stand for test and initial value of surface potential, respectively.

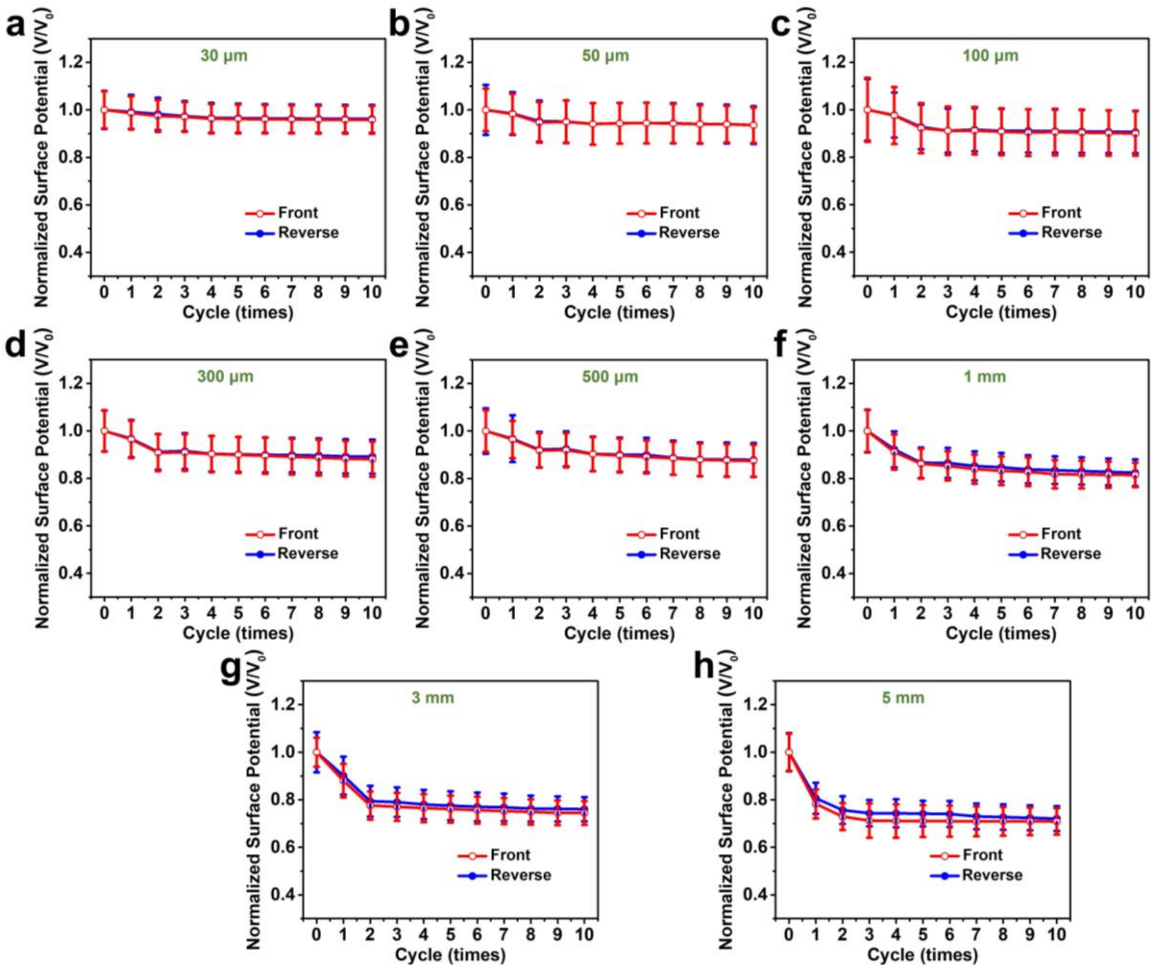

Figure S8. Surface potential characteristics of the SL-PTFE with a series of thicknesses over frontal contact with water 10 times; V and V_0 stand for test and initial value of surface potential, respectively.

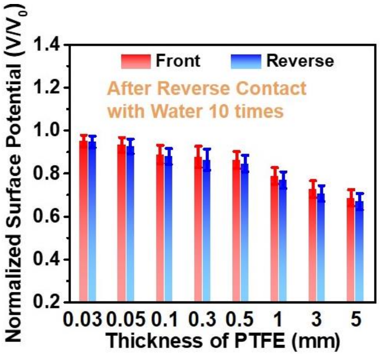

Figure S9. Surface potential characteristics of the SL-PTFE with a series of thicknesses after reversed contact with water 10 times; V and V_0 stand for test and initial value of surface potential, respectively.

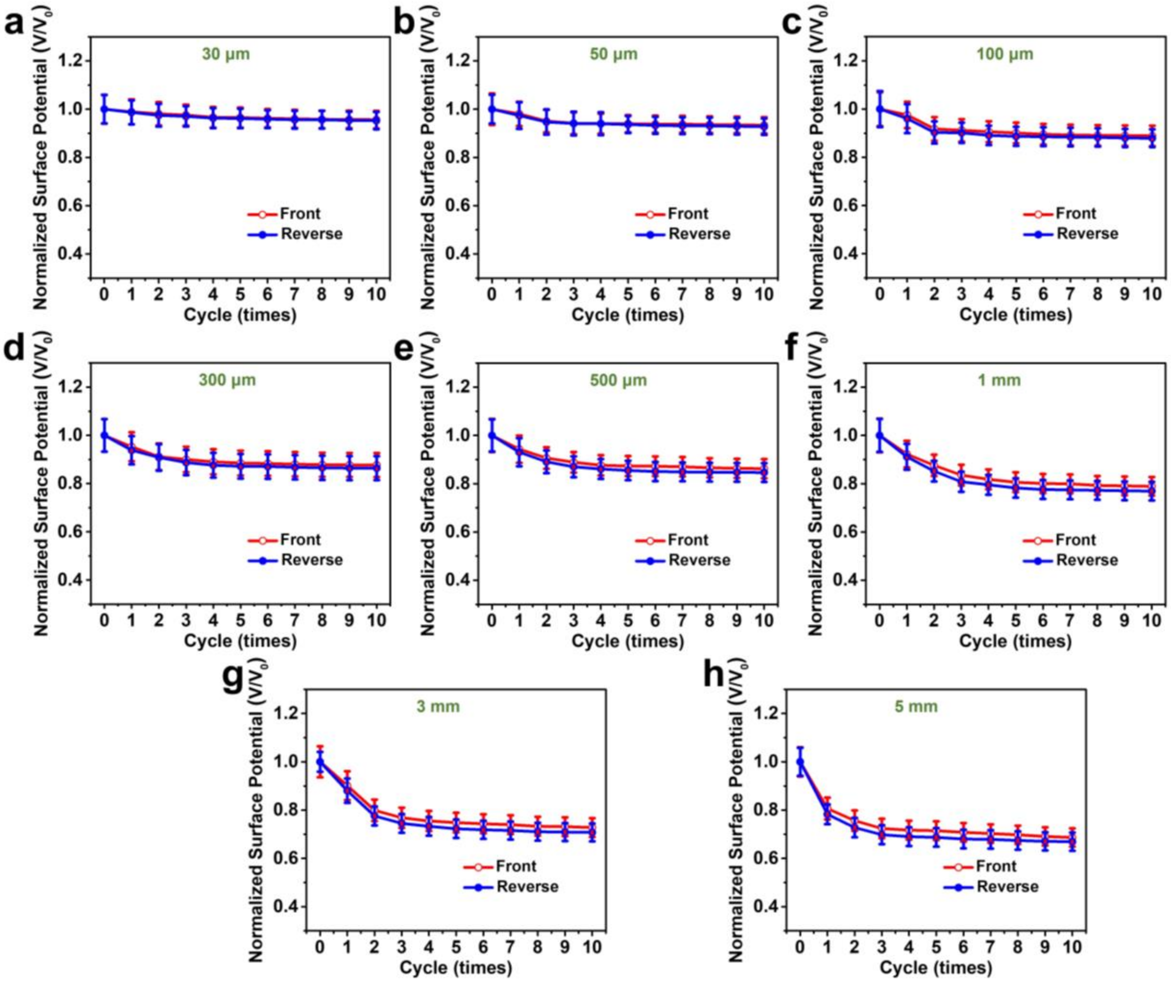

Figure S10. Surface potential characteristics of the SL-PTFE with a series of thicknesses over reversed contact with water 10 times; V and V_0 stand for test and initial value of surface potential, respectively.

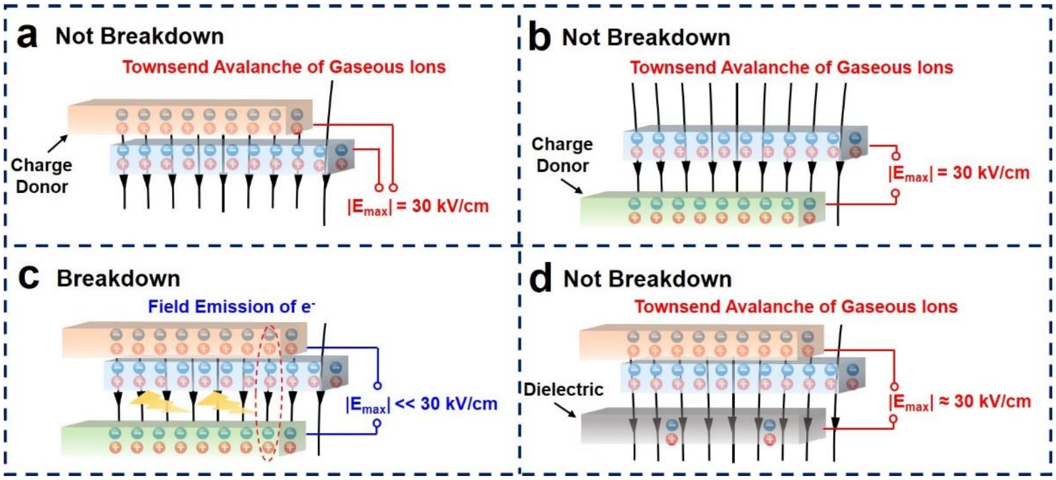

Figure S11. Breakdown electric field of the SL-PTFE under different states.

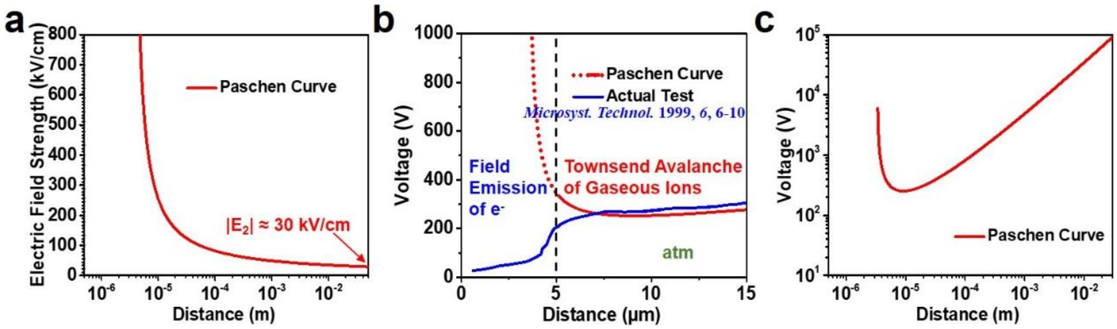

Figure S12. a) Electric field strength curve of Paschen's law, **b)** voltage curve of air breakdown, **c)** voltage curve of Paschen's law; atm stands for atmosphere.

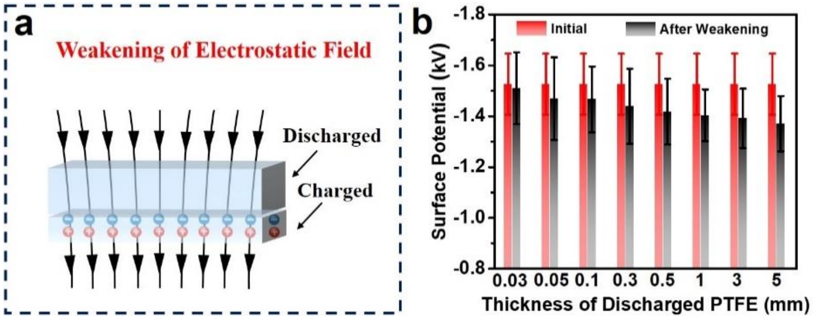

Figure S13. a) Schematic and **b)** surface potentials of the SL-PTFE being covered with various discharged PTFE of different thicknesses.

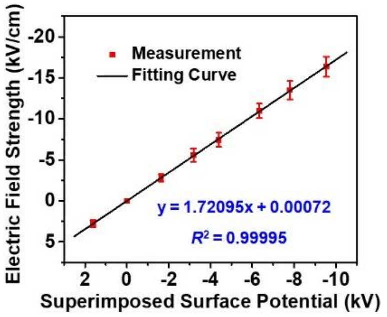

Figure S14. Corresponding relationship between the superimposed surface potential and electric field strength of the ML-PTFE.

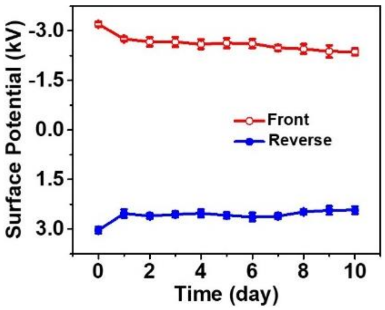

Figure S15. Surface potential characteristic of the DL-PTFE over 10 days.

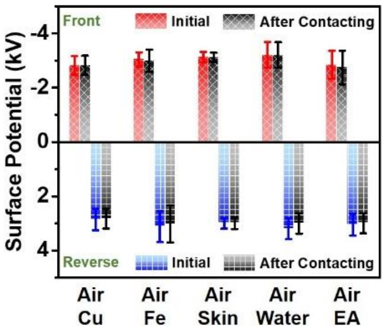

Figure S16. Initial and remaining surface potentials of the DL-PTFE after contact with various charge donors on the reverse side.

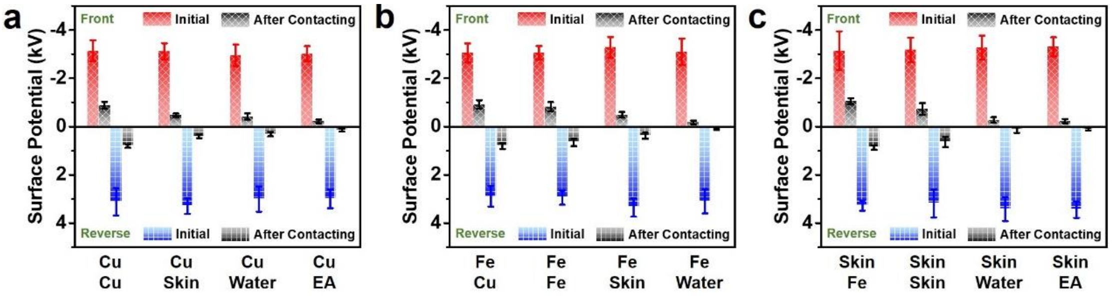

Figure S17. a-e) Initial and remaining surface potentials of the DL-PTFE after contact with various charge donors on the front side and reverse side simultaneously.

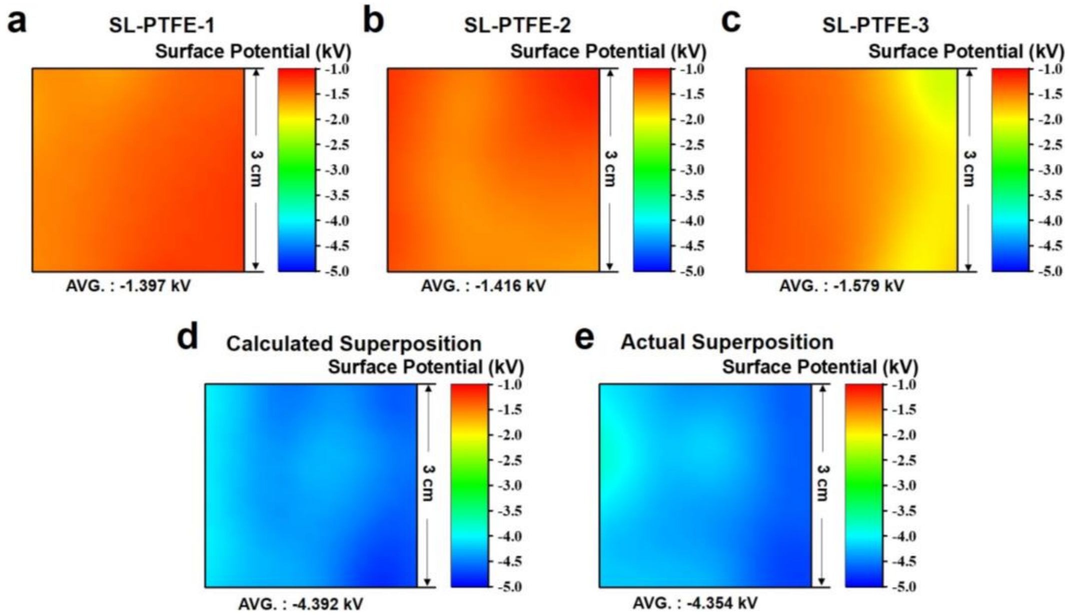

Figure S18. Surface potential distributions of the **a)** SL-PTFE-1, **b)** SL-PTFE-2, **c)** SL-PTFE-3, and **d)** calculated superposition and **e)** actual superposition of the SL-PTFE-1, SL-PTFE-2, and SL-PTFE-3.

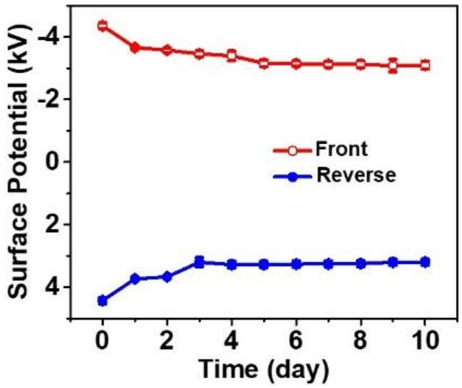

Figure S19. Surface potential characteristic of the TL-PTFE over 10 days.

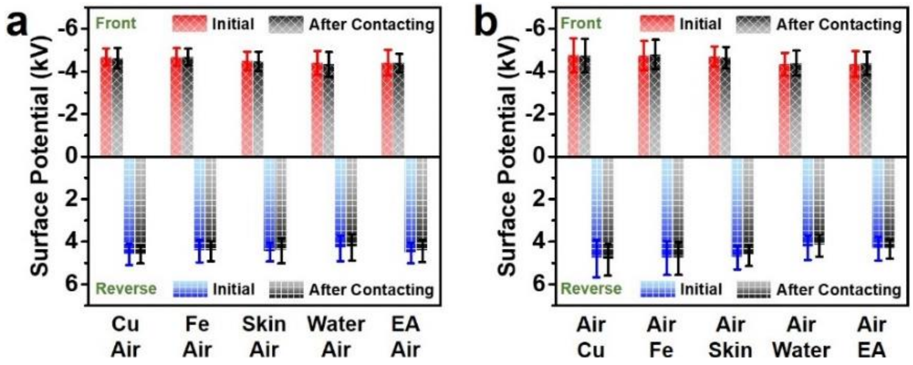

Figure S20. Initial and remaining surface potentials of the TL-PTFE after contact with **a)** various charge donors on the front side and **b)** reverse side separately.

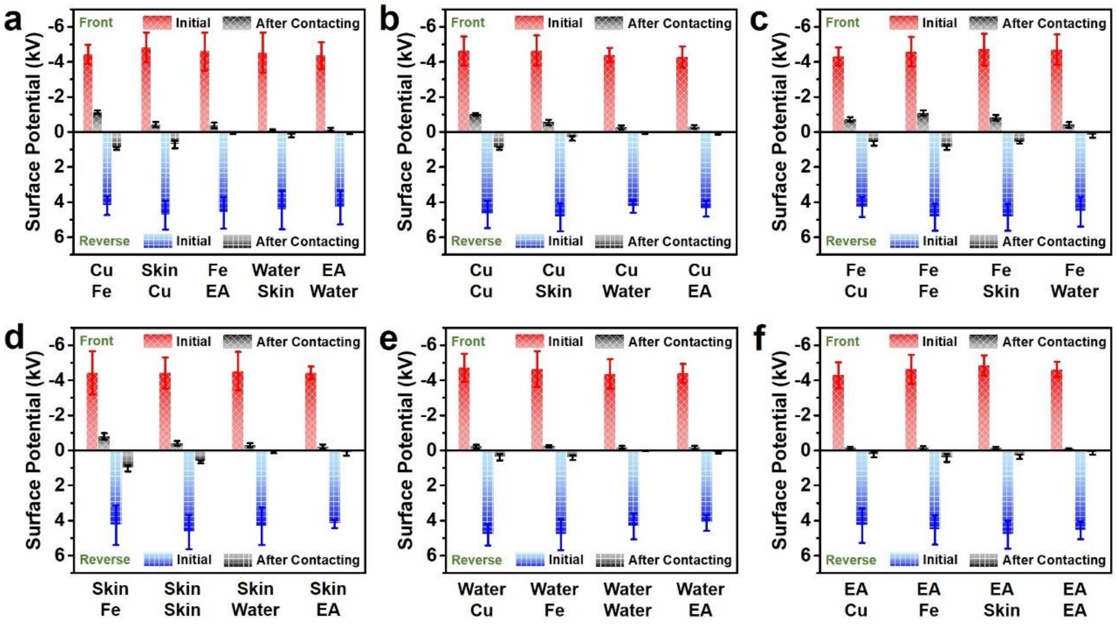

Figure S21. a-f) Initial and remaining surface potentials of the TL-PTFE after contact with various charge donors on the front side and reverse side simultaneously.

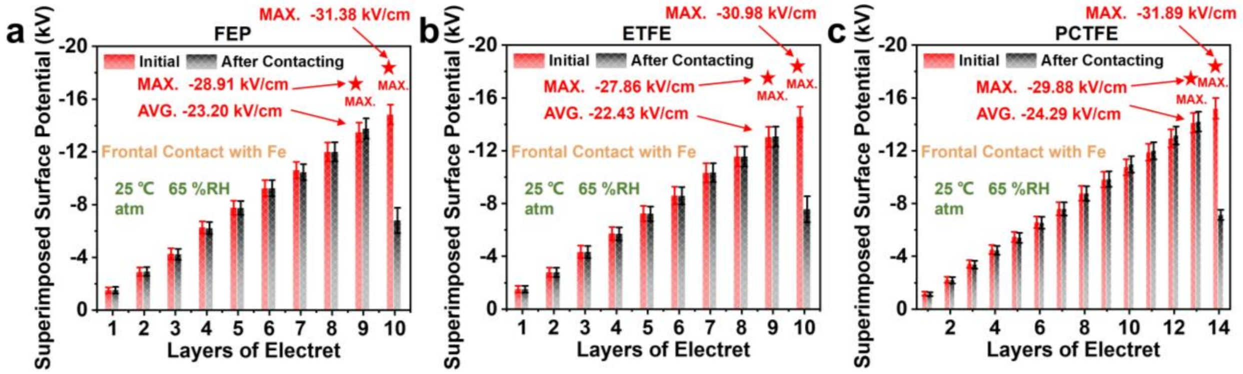

Figure S22. Superimposed surface potentials of the a) ML-FEP, b) ML-ETFE, and c) ML-PCTFE with gradually increasing layers before and after frontal contact with Fe; MAX., AVG., and atm stand for maximum, average, and atmosphere, respectively.

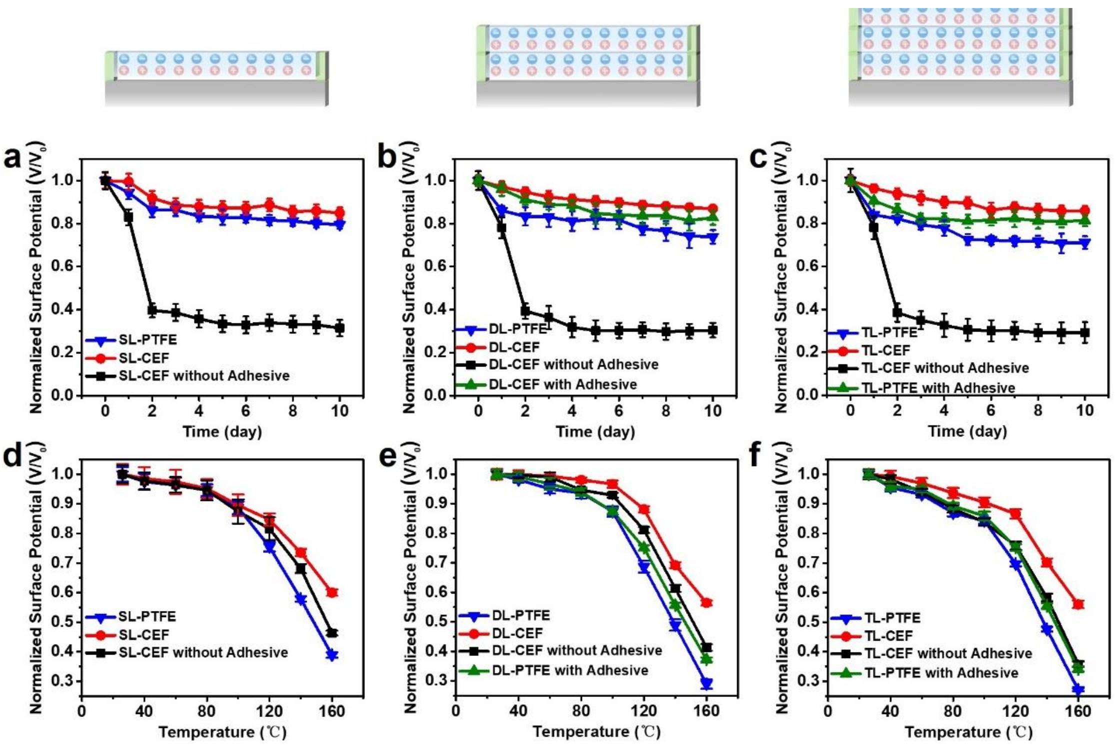

Figure S23. Normalized surface potentials of various electret films **a-c)** over 10 days and **d-f)** after annealing at a series of temperatures for 30 min; V and V_0 stand for test and initial value of surface potential, respectively.

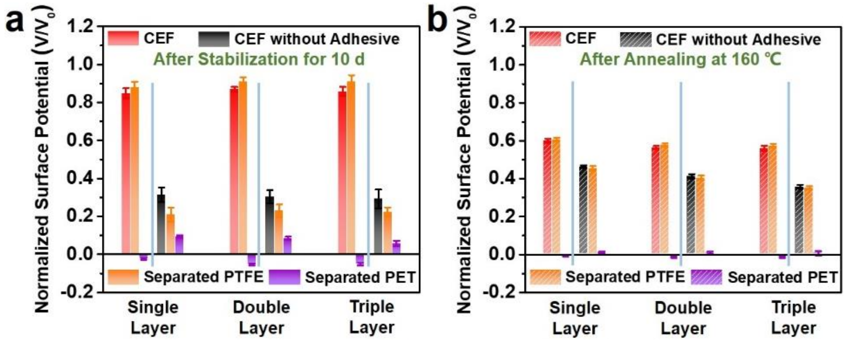

Figure S24. Normalized surface potential of various films **a)** after stabilization for 10 days and **b)** after annealing at 160 ^{o}C for 30 min; V and V_0 stand for test and initial value of surface potential, respectively.

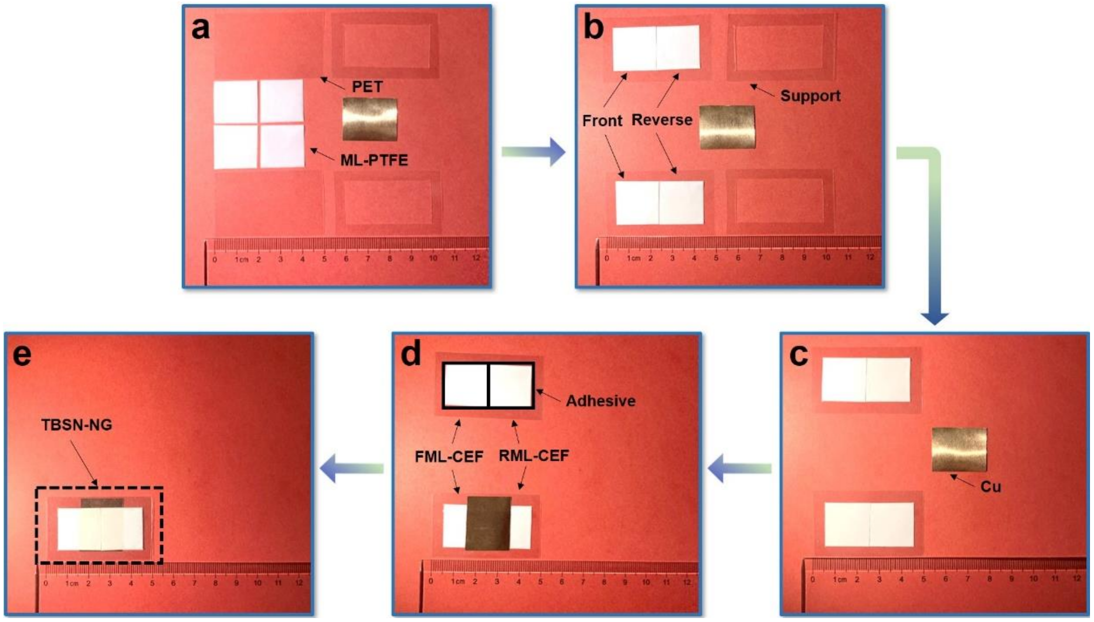

Figure S25. a-e) Digital images of fabricating process of the TBSN-NG.

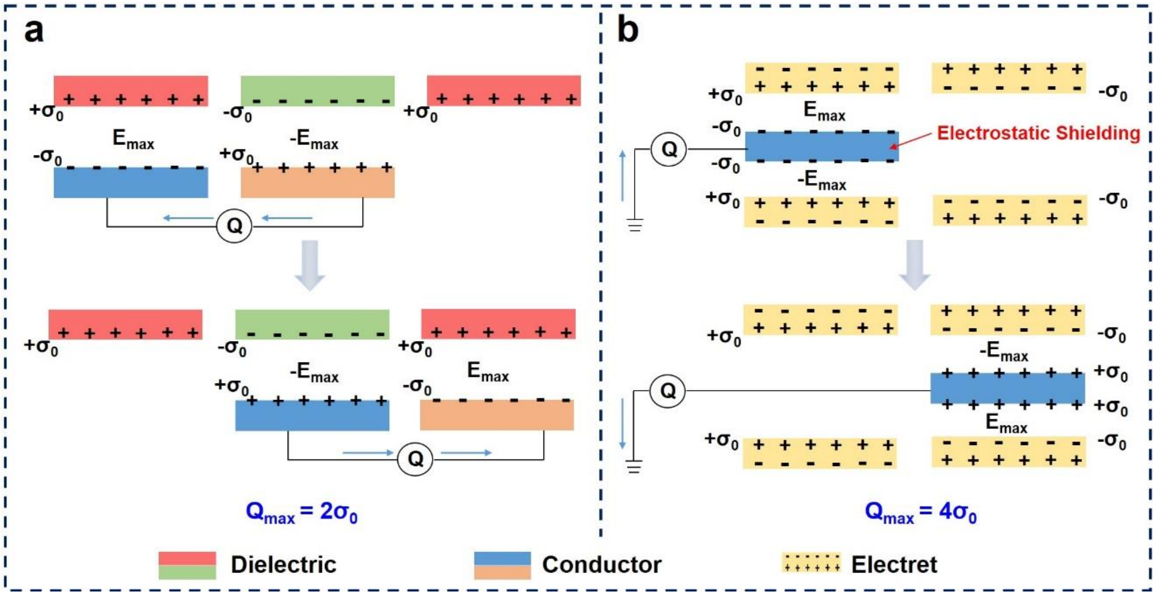

Figure S26. Working states of non-contact nanogenerators with **a)** one-sided and **b)** two-sided electrostatic induction structure during periodic sliding cycles.

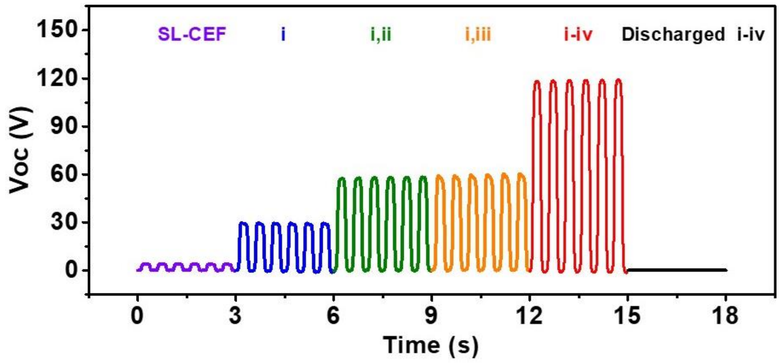

Figure S27. Dynamic output open-circuit voltage curves of six types of NGs being built by different CEFs at a sliding speed of ~8 cm/s.

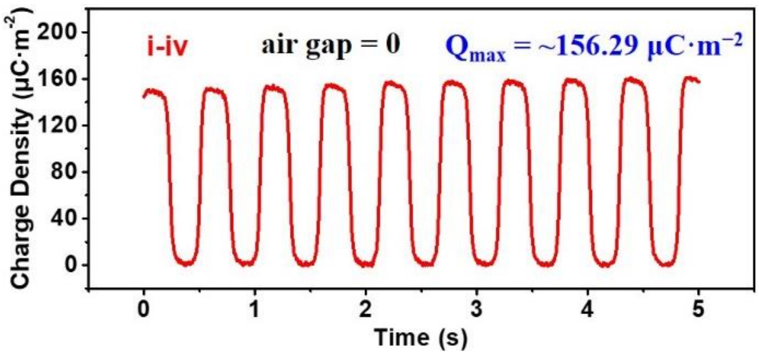

Figure S28. Dynamic output charge curve of the TBSN-NG in the contact mode.

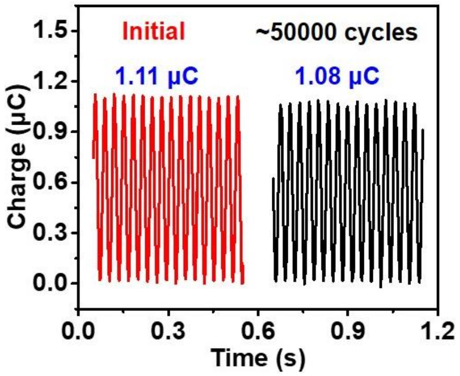

Figure S29. Output charge curves of the TBSN-RDNG at ~300 rpm before and after ~50000 cycles.

Table S1. Comparison of the Surface Potential and Electric Field Strength

Sample	Surface Potential (kV)	Electric Field Intensity (kV/cm)
PTFE (front)	-1.635 ± 0.276	-2.81 ± 0.43
PTFE (reverse)	1.618 ± 0.276	2.79 ± 0.42

Table S2. Comparison of the Surface Potential of the SL-PTFE after Contact with Various Charge Donors on Both Sides Simultaneously

Charge Donors		Remaining Surface Potential (kV)	
Front	Reverse	Front	Reverse
Cu	Fe	34.8%	36.4%
Skin	Cu	21.8%	21.9%
Fe	EA	10.3%	9.6%
Water	Skin	4.0%	3.8%
EA	Water	0.5%	0.4%

Table S3. Comparison of the Charge Stability among Various Electret Films								
	After Stabilization for 10 days Remaining Surface Potential (kV)				After Annealing at 160 °C for 30 min Remaining Surface Potential (kV)			
	PTFE	CEF	CEF without Adhesive	PTFE with Adhesive	PTFE	CEF	CEF without Adhesive	PTFE with Adhesive
Single Layer	79.5%	84.9%	31.4%		38.9%	60.0%	46.3%	
Double Layer	73.9%	86.9%	30.5%	82.8%	29%	56.4%	41.3%	37.2%
Triple Layer	71.1%	85.9%	29.2%	81.4%	27.1%	56.0%	35.8%	34.2%

Table S4. Comparison of the Non-Contact Nanogenerator			
Sample	Gap (mm)	Charge Density (μC·m^{-2})	Core
This Work	0.5	132.6	ML-CEF
Energy Environ. Sci. 2021, 14: 1004	1	87.3	C_{60}-Containing Block Polyimide
Nat. Commun. 2021, 12: 4689	0.35	71.5	Extra Circuit
Nano Energy 2021, 81: 105625	< 3	> 20.3	Rabbit Hair Brushes
ACS Energy Lett. 2020, 5: 3005−3011	0−1	25	Fish Bladder Film
Nano Res. 2020, 13: 1903–1907	1	1.6	Nanostructured FEP
Adv. Energy Mater. 2020, 10: 2000064	1.5	16.3	PTFE Stripes
